# Systematic Review of Pediatric Functional Gastrointestinal Disorders (Rome IV Criteria)

**DOI:** 10.3390/jcm10215087

**Published:** 2021-10-29

**Authors:** Angharad Vernon-Roberts, India Alexander, Andrew S. Day

**Affiliations:** Department of Paediatrics, University of Otago Christchurch, Christchurch 8011, New Zealand; alein924@student.otago.ac.nz (I.A.); Andrew.day@otago.ac.nz (A.S.D.)

**Keywords:** prevalence, functional GI, gut disorders, Rome IV

## Abstract

Functional gastrointestinal disorders (FGID) are common among children and may cause a significant symptom burden. The Rome criteria are symptom-based guidelines for the assessment of FGID among children and adults. The aim of this systematic review was to estimate the prevalence of FGID utilizing the revised Rome IV criteria. Nine health databases were searched. The inclusion criteria were: prospective FGID prevalence data using the Rome IV criteria for children up to 18 years, and the exclusion criteria were: cohorts with known gastrointestinal or organic conditions. The data were presented as a percentage of children experiencing at least one FGID, as well as in individual categories. The searches identified 376 papers, with 20 included in the final analysis, providing a pooled cohort of 18,935 children. The median prevalence of FGID for children aged up to four years was 22.2% (range 5.8–40%), and aged four–eighteen years was 21.8% (range 19–40%). The most common FGID for children aged 0–12 months was infant regurgitation, the most common FGID for those aged 13–48 months were functional constipation and cyclic vomiting, and, for those aged over four years, functional constipation, functional dyspepsia, and irritable bowel syndrome. This reported overall incidence of FGID may be used as a benchmark of normative data among the general population and comparative data for those with comorbid disease.

## 1. Introduction

Functional gastrointestinal disorders (FGID) are common conditions among children and are characterised by recurring GI symptoms that are not attributable to structural or biochemical abnormalities or as a comorbidity of organic disease [1]. FGID may be caused by various factors, such as disturbances in gut motility, visceral hypersensitivity, mucosal and immune function, gut microbiota, and central nervous system processing [2]. FGID in children lead to a significant symptom burden with associated psychological distress, reduced quality of life, school absenteeism, greater health care expenditure, and missed work for parents [3]. Furthermore, FGID in childhood is also linked to the progression of the disorder into adulthood, such that 25% of children who present with recurrent abdominal pain may subsequently develop irritable bowel syndrome (IBS) as an adult [4]. 

The diagnosis of FGID may be made using the Rome criteria, which are symptom-based guidelines developed using scientific evidence and clinical experience [5]. The most recent iteration is Rome IV, which has paediatric versions available for toddlers (up to four years of age) [6] and children and adolescents (aged four to eighteen years) [5]. 

The aim of this systematic review was to assess the rates of FGID reported among the two age groups using the Rome IV criteria, focusing on cohorts from the general population with no underlying organic disease or presenting GI symptoms in order to provide a benchmark for comparative studies.

## 2. Materials and Methods

### 2.1. Eligibility Criteria

The following eligibility criteria were required to be met: to include data on children up to 18 years of age, report an overall cohort rate with at least one FGID, score FGID according to Rome IV criteria (unmodified except for translation), the cohort to have no known pre-existing gastrointestinal symptoms or organic conditions, and studies must have undergone prospective data collection. 

### 2.2. Information Sources

The systematic review protocol, search strategy, implementation and reporting were performed using the Preferred Reporting Items for Systematic Reviews and Meta-Analyses (PRISMA) guidelines [7] (Checklist S1). The following databases were searched: Medline, Embase, Cumulative Index to Nursing and Allied Health Literature (CINAHL), PsychInfo, Cochrane database, Scopus, Joanna Briggs Institute, ProQuest, and OpenGrey. 

### 2.3. Search Strategy

The individual search strategies are included (Appendix A), but the main terms included were related to: Rome IV, functional gastrointestinal disorders, and children. Additional search limits were applied for the year 2016 onwards to reflect when the year the Rome IV criteria were published.

### 2.4. Selection and Data Collection Process

All identified papers were synthesised into a database, duplicates removed, and the remaining titles examined by two reviewers (A.V.-R. and I.A.) to identify those satisfying inclusion criteria and being relevant for a full text review. Disputes were resolved by discussion between three reviewers (A.V.-R., I.A., and A.S.D.). All relevant articles were read in full text by two reviewers (A.V.-R. and I.A.). 

### 2.5. Data Items

Outcome data were sought for the overall percentage of children experiencing at least one FGID, as well as the percentage experiencing FGID according to the individual Rome IV categories. If studies reported an overall percentage of children experiencing one FGID, it was included in the systematic review, and individual category data were collected where available. 

### 2.6. Effect Measures

Effect measures for FGID are presented as the number and/or percentage of children experiencing at least one FGID, and the number and/or percentage of children experiencing FGID within the Rome IV categories.

### 2.7. Synthesis Methods

Data were extracted from retrieved records and entered in to a spreadsheet to record study details, cohort descriptives, and all data reporting overall FGID as well as results for each Rome IV category, where available. Missing data are presented as ‘not reported’. If papers presented data on different age cohorts, these were presented and assessed separately according to the age group. If papers included a case control study, only data for the control group were presented and assessed. Summary statistics were used to provide an overall and category prevalence estimate of FGID among the cohorts with data presented in tables and box plots. In the age group of children up to four years, FGID category data were divided in to the age ranges of infant (0–12 months) and toddler (13–48 months) where possible.

### 2.8. Risk and Reporting of Bias Assessment

The assessment of methodological strengths, results, and relevance of included studies were determined by standards for critical appraisal of research literature relating to prevalence and incidence of a health problem [8]. Papers were assessed against criteria to assess the validity of study methods, results interpretation, and applicability of the results, and assigned a score, as taken from Loney et al. [8]. This process is described in Supplementary Material (Table S1).

### 2.9. Certainty Assessment

Descriptive data only are presented in this review; certainty of results will be discussed in the context of bias assessment and overall results.

## 3. Results

### 3.1. Study Selection

Three hundred seventy-six publications were identified from the searches (Appendix A), and 20 met the inclusion criteria of reporting the prevalence of FGID among children using the Rome IV criteria (Figure S1). 

### 3.2. Results of Individual Studies 

The characteristics of each included study were examined alongside general cohort information (Table 1). Nine studies were from countries in South America, eight from Europe, two from Asia, and one from North America. For those papers reporting from the same country, it was confirmed that each cohort was specific to that research with no reporting overlap between studies. Five (25%) studies recruited participants from healthcare clinics, five (25%) from well-baby/child clinics, five (25%) recruited in schools, one (5%) via an internet survey, and four (20%) did not state this information. The Rome IV assessments were completed by a number of groups: 2 (10%) researchers, 1 (5%) physicians, 13 (65%) by family members, with 4 (20%) studies providing insufficient information. The combined study populations reported FGID in cohorts ranging in size from 65 to 2751 children, representing a pooled cohort of 18,935 children, with approximately half the overall cohort being female. 

### 3.3. Results of Synthesis

#### 3.3.1. Prevalence of Overall FGID

The prevalence of FGID from the included studies were stratified according to the Rome IV version for children aged up to four years (Table 2) and from four to eighteen years (Table 3). 

The overall prevalence of children with at least one FGID were similar between the age groups, with a median 22.2% (inter-quartile range (IQR) 15.7, range 5.8–40%) for the cohort aged up to four years, and a median 21.8% (IQR 6.3, range 19–40%) for the cohort aged four years and over (Figure S2). Five papers reported specifically on the number of children under four years with more than one FGID, with 0.4–4.5% having two FGID, [12,18,22] and 0.1–0.6% having more than two or multiple FGID [22,27]. Among children over four years, 4% had multiple FGID [14]. 

#### 3.3.2. Prevalence of Sub-Category FGID

Rome IV is also divided in to sub-categories, with seven FGID categories for children aged up to four years and ten categories for those over four years. 

##### Infant/Toddler Categories

**Figure 1:**
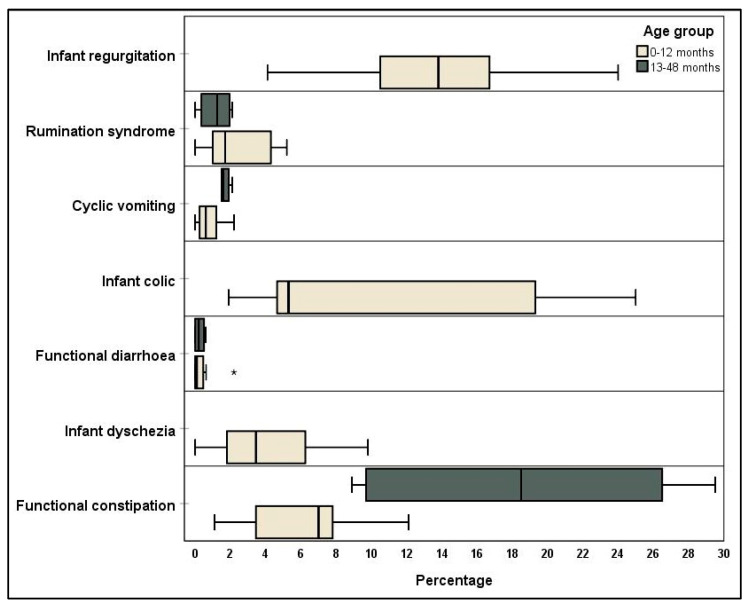
Percentage incidence of specific Rome IV criteria for children up to the age of four years. *: outlier.

The seven categories for infants (age 0–12 months) and toddlers (13–48 months) are further divided, whereby infants can satisfy criteria for any FGID, and toddlers generally for the categories of cyclic vomiting, functional diarrhoea, and functional constipation [6]. The most prevalent FGID from the included studies that had reported data divided in to appropriate age groups were infant regurgitation and functional constipation, and, in the toddler age group, were functional constipation and cyclic vomiting (Figure 1). 

##### Children/Adolescent Categories

**Figure 2:**
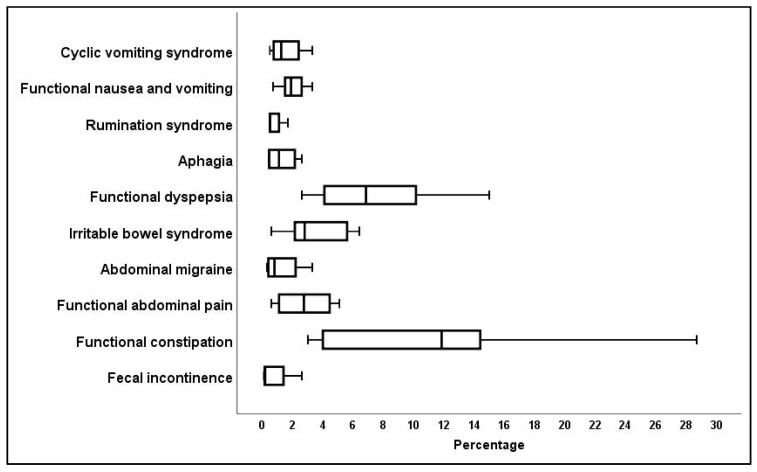
Percentage incidence of specific Rome IV criteria for children over the age of four years.

The most prevalent of the ten FGID categories among the studies presenting data on children over the age of four years were shown to be functional constipation, functional dyspepsia, and irritable bowel syndrome (IBS) (Figure 2). 

#### 3.3.3. FGID Sub-Types

The Rome IV criteria for children over the age of four are further divided to provide additional clarification of the FGID sub-types [5]. The classification of functional nausea and vomiting disorders into categories of functional nausea and functional vomiting showed that, of the four papers that classified their data in this category, the percentage of children experiencing functional vomiting was up to 2.2% greater than those experiencing functional nausea [19,20,25,26]. Five papers further classified functional dyspepsia into the categories of post-prandial distress syndrome (PPDS) and epigastric pain syndrome (EPS), showing that the percentage of children who experienced PPDS was up to 6.8% greater than those experiencing EPS [19,20,25,26,28]. The category of IBS was classified with the sub-types of IBS with constipation, IBS with diarrhoea, IBS with constipation and diarrhoea (mixed type), and unspecified IBS. Five papers divided their IBS sub-types, with the main finding being that up to 8.8% more experience constipation-predominant IBS than diarrhoea-predominant IBS [14,15,20,25,26].

### 3.4. Risk of Bias of Studies

Of the twenty papers, 9 (45%) were published conference abstracts and 11 (55%) full manuscript publications. The risk of bias for each study was assessed according to specific criteria and a total score calculated to a maximum of eight (Table S1). The total scores showed that 13 (65%) of the studies satisfied more than half of the quality assessment criteria. The sources of bias most evident were for location sampling and the reporting of data (either incomplete descriptive statistics or missing data). The location sampling bias was from studies either recruiting from one single site or not stating their sampling location. Of those studies with missing data, three account for this as the papers concentrated on the incidence of functional constipation only but did report an overall number with at least one FGID [16,17,21]. Three papers only reported on those categories in the infant (0–12 month) age group for those that had ‘infant’ in front of the category title, although this age cohort may satisfy all the criteria [10,18,24]. One paper stated that they included data for the ‘main’ FGID categories [23].

### 3.5. Certainty of Data

The limitations highlighted in the bias assessment were not considered to impair the generalisability of the overall results. All the studies reported data on children from the general population with no comorbid conditions, and the pooled data provide a large population from which to draw conclusions. The data on individual categories may be limited by some instances of missing data, but the percentages are not inflated by this factor as category diagnostic criteria are still required to be met. 

## 4. Discussion

This systematic review has provided a pooled incidence of the percentage of children in the general population experiencing FGID according to the Rome IV criteria. The rates of FGID were similar for different age groups of children and categories identified that are the most prevalent. 

While previous systematic reviews of FGID utilising Rome III are available for broad comparison with this review, they are not directly comparable due to between-version changes [5,29]. The Rome III criteria identified 27–38% with at least one FGID [30] in the infant/toddler cohort, but this report showed a lower pooled incidence, with 22% having at least one FGID, but a greater range (5–40%). The most common FGID categories using Rome III were infant regurgitation and functional constipation [30]; however, this review divided the infant/toddler categories and reports a greater range of infant colic (1–25%) than Rome III (1–5%) [30]. The revisions to this category in Rome IV to remove arbitrary crying limits and focus on factors shown to cause distress in parents, such as the prolonged, hard-to-soothe nature of the crying behaviour, may be attributable to this increase and may also account for the greater range of FGID overall [6,29]. The greater prevalence of constipation shown in the toddler versus infant group highlights the importance of separating out the infant/toddler data where possible. The wide variation in the confidence intervals for FGID among this age cohort may indicate that prevalence estimates are less certain and that the Rome IV diagnostic criteria may not yet be specific enough in this age group.

In the child/adolescent cohort, those experiencing at least one FGID criterion were 9–29% according to Rome III [31], compared to 21% (range 19–40%) in this review. This increase may be attributable to the new category of functional nausea and vomiting, although the ranges of these particular disorders were relatively low in the current report. The incidence of FGID among adults is approximately 40%, so the inclusion of studies with a greater proportion of children at the upper end of the age limit of 18 years may contribute to this higher upper range [2]. Both the Rome III and Rome IV criteria show their highest FGID category being functional constipation [31] and, while Rome III also reports high rates of cyclic vomiting, in this review, the second highest incidence was of functional dyspepsia. The functional dyspepsia category underwent major revisions between Rome III and IV and was expanded to include postprandial fullness, early satiation, and epigastric pain [29]. This may have changed the reporting status of functional dyspepsia, and the addition of the functional nausea category may have reduced the reporting of cyclic vomiting syndrome and subsequently increased the overall FGID incidence.

The Rome IV criteria no longer require there to be an exclusion of organic disease in recognition of the fact that comorbid FGID may be present alongside pre-existing conditions [5,29]. However, by excluding papers where children had a co-existing organic condition, we have provided a sample representative of the general population and prevented the inflation of the overall incidence of FGID. Children with organic disease have been shown to have a significantly higher prevalence of FGID than controls, for example, those with epilepsy [9], Familial Mediterranean Fever [14], and Henoch–Schönlein purpura [15]. Comorbid FGID may also be present with organic gastrointestinal conditions, such as inflammatory bowel disease [32,33] and coeliac disease [34], as well as other comorbid conditions, such as joint hypermobility [35] and obesity [36]. However, these findings are not universal [37,38] and may lead to speculation that the nature and cause of functional symptoms may not be well defined for children with co-existing conditions.

Variation in who completed the Rome IV assessment may have introduced reporting bias as, while having researchers or clinicians complete the assessment may be considered more objective than others, there is increasing recognition that children self-reporting is the gold standard for clinical health status indicators [39,40]. However, the use of the standardised Rome IV reporting tool that includes an objective child report and parent proxy report should minimise this effect. The visual inspection of the four papers with researcher/clinician completion identified no trends towards over- or underestimation.

### 4.1. Strengths

This review used a comprehensive search strategy that included grey literature and published conference abstracts, thereby minimising publication bias. The inclusion criteria of studies including prospectively collected data ensured diagnostic accuracy and reduced the possibility of recall bias. 

### 4.2. Limitations

The inclusion criteria requirement of studies reporting numbers experiencing at least one FGID allowed the inclusion of papers that reported on one specific category, such as functional constipation. This led to a small number of studies having missing data, although it provided an overall pooled estimation of FGID. 

## 5. Conclusions

This review provides an overview of the reported incidence of FGID using the Rome IV criteria among children. It has enabled a direct comparison with similar reviews using the Rome III criteria and highlighted the reasons for variations in the data. In providing an overall incidence of FGID among the general population, this review may be used as a benchmark of normative data for future cohorts utilising the Rome IV criteria in the general population, as well as comparative data for those with comorbid disease. 

## Figures and Tables

**Table 1:** Characteristics of studies included in data extraction process.

Author	Year	Country	Population Sampling	Recruitment Site	Rome IV Completion	Cohort Number	Age	Gender
* Aydemir [9]	2020	Turkey	Matched controls	-	Researcher	78	-	53% F
Beser [10]	2019	Turkey	Random sampling	Regional out-patient clinics	Physician	2383	Range 0 to 12 m	-
Chanis [11]	2019	Panama	Random sampling	One well-child clinic	Parent	65	Mean 6.9 m (SD 3.9)	44.6% F
Chew [12]	2021	Malaysia	Whole population	Regional well-baby clinics	Parent	534	Mean 6.8 m (SD 3.4) Range 1 to 12 m	54.1% F
Chikunov [13]	2019	Russia	Random sampling	One University clinic	Unclear	300 0–6 m: 180 7 m−4 y: 120	Range 0 to 4 y	Age 0–6 m 38.9% F Age 7 m−4 y 58.3% F
* Ekinci [14]	2019	Turkey	Matched controls	-	Child/family	100	Mean 9.7 (SD 3.6) Range 4 to 18 y	34% F
* Ekinci [15]	2019	Turkey	Matched controls	-	Unclear	78	Median 9.2 y (IQR 4.5–17.7) Range 4 to 18 y	33% F
Jativa-Marino [16]	2019	Ecuador	Random sampling	Regional schools	Child	951	Mean 11.2 y (SD 1.8)	39.3% F
Llanos-Chea [17]	2019	Colombia	Random sampling	Regional well-child clinics	Parent	815	Mean 17.6 m Range 1 to 48 m	-
Ozdemir [18]	2018	Turkey	Random sampling	One out-patient clinic	Unclear	481	Range 0 to 12 m	-
Robin [19]	2018	United States	Random but mothers only	Internet survey	Mother	1255 0–12 m: 58 1–3 y: 238 4–18 y: 959	Mean 8.35 y Range 0 to 18 y	53.2% F
Saps [20]	2018	Colombia	Random sampling	Regional schools	Child	3567	Mean 13.7 y (SD 2.4)	56.5% F
Saps [21]	2020	Colombia	Random sampling	Regional out-patient clinics	Parent	1334 0–12 m: 389 13–48 m: 945	Mean 24.4 m (SD 15)	51% F
Steutel [22]	2020	Belgium Italy Netherlands	Random sampling	Regional well-child clinics	Researcher	2751 0–12 m: 1698 13–48 m: 1053	Age 0–1 m: Median 4.1 m (IQR 2.1–7.5) Age 13–48 m: Median 26.4 m (IQR 18.7–38.7)	Age 0–1 m 47.4% F Age 13–48 m 48.2% F
Velasco-Benitez [23]	2018	Colombia	Random sampling	One school	Child	330	Range 10 to 18 y	50.3% F
Velasco-Benitez [24]	2019	Colombia	Random sampling	-	Parents	1298	Mean 24.9 m (SD 15.1)	51.2% F
Velasco-Benitez [25]	2020	Colombia	Random sampling	Regional schools	Child/family	1497	Mean 13.4 y (SD 2.1) Range 10 to 18 y	71% F
Velasco-Benítez [26]	2021	Colombia	Random sampling	Regional schools	Child	465	Mean 12.9 y (SD 1.3) Range 10 to 18 y	48.2% F
Vlad [27]	2019	Romania	Random sampling	Regional family clinics	Unclear	308 0–12 m: 174 1–3 y: 134	Mean 12.2 m Range 0 to 3 y	-
Zeevenhooven [28]	2019	Netherlands	Selected cohort	Follow up clinic	Child/family	102	Median 17 y (IQR 17–17)	56% F

m = months, y = years, F = female, - = not reported, * = data on controls presented only.

**Table 2:** Prevalence of overall and sub-category FGID among cohorts of children under the age of four years.

First Author	Age Range	Cohort Number	% with at Least One FGID	Infant Regurgitation	Rumination Syndrome	Cyclic Vomiting	Infant Colic	Functional Diarrhoea	Infant Dyschezia	Functional Constipation
Beser [10]	0–12 m	2383	35.1	13.4	-	-	19.2	-	9.8	-
Chanis [11]	0–12 m	65	40	21	0	0	25	0	2.3	7.7
Chew [12]	1–12 m	534	14.6	10.5	1.7	0	1.9	0.3	1.3	1.1
Chikunov [13]	0–6 m	180	23	5.6	1	0.6	19.4	2.2	5.6	3.9
	7 m−4 y	120	18	0.8	0.8	3.3	1.65	6.7	0.8	31.7
Llanos-Chea [17]	1–48 m	815	21.2	-	-	-	-	-	-	9.6 ^#^
Ozdemir [18]	0–12 m	481	5.8	4.1	-	-	2.2	-	0.62	-
Robin [19]	0–12 m	58	37.9	24	1.7	1.7	5.2	0	0	12.1
Robin [19]	13–36 m	238	21.4	*	2.1	2.1	*	0	*	18.5
Saps [21]	0–12 m	389	30.6	-	-	-	-	-	-	7.7 ^#^
	13–48 m	945		-	-	-	-	-	-	26.5 ^#^
Steutel [22]	0–12 m	1698	24.7	13.8	4.3	0.7	4.2	0.1	4.0	3.0
	13–48 m	1053	11.3	*	0	1.5	*	0.6	*	9.7
Velasco-Benitez [24]	0–12 m	1298	32.1	16.7	-	-	5.4	-	2.9	7.9
	13–48 m			*	1.8	1.7	*	0.4	*	29.5
Vlad [27]	0–12 m	174	21.4	15.5	5.2	0.5	5.1	0	6.9	6.3
	13–48 m	134		*	0.7	1.5	*	0	*	8.9

All data presented as %; -: not reported, * = 12 month + categories assessed only, # constipation categories reported only.

**Table 3:** Prevalence of overall and sub-category FGID among children/adolescents over the age of four years.

			Functional Nausea and Vomiting Disorders				Functional abdominal Pain disorders				Functional Defecation Disorders	
First author	Cohort number	% with at least one FGID	Cyclic vomiting	Functional nausea and vomiting	Rumination syndrome	Aerophagia	Functional dyspepsia	Irritable bowel syndrome	Abdominal migraine	Functional abdominal pain	Functional constipation	Fecal incontinence
Aydemir [9]	78	19.2	0	2.6	0	0	0	6.4	0	1.3	6.4	2.6
Ekinci [14]	100	19	0	0	0	0	10	3	0	4	4	0
Ekinci [15]	78	19.2	0	0	0	0	10.3	2.6	0	5.1	3.8	0
Jativa-Marino [16]	951	22.3	-	-	-	-	-	-	-	-	14.4 ^#^	-
Robin [19]	959	25	2	1.9	0	2.6	7.6	5.1	1.1	3.1	14.1	0.2
Saps [20]	3567	21.2	0.5	0.7	0.5	0.5	3	2.3	0.5	2.4	10.7	0.1
Velasco-Benítez [26]	465	20.8	3.3	3.3	1.7	1.7	6.1	0.6	3.3	0.6	28.7	0
Velasco-Benitez [23]	330	40.0	-	-	-	-	5.2	6.1	-	4.9	22.7	-
Velasco-Benitez [25]	1497	22.7	1.5	1.5	0.5	0.4	2.6	2.0	0.3	0.9	13	0
Zeevenhooven [28]	102	27	1	0	0	0	15	0	0	0	3	0

All data presented as %; - = not reported, # = constipation categories only reported.

## Data Availability

Data may be obtained from the corresponding author on submission of written request.

## Supplementary Materials

The following are available online at https://www.mdpi.com/article/10.3390/jcm10215087/s1, Figure S1: PRISMA guideline flow chart of the article selection process, Figure S2: Comparison of overall percentage prevalence in each age group experiencing at least one FGID (*: outlier). Table S1: Risk of bias assessment for included articles, Checklist S1:PRISMA item checklist.

## Appendix A. Search Results for Health Literature Databases

**Table A1:** MEDLINE.

Search Line	Search Statement	Results
1	Rome IV.mp.	431
2	limit 1 to yr = “2016 -Current”	427
3	functional gastro *.mp.	3197
4	GI disorder.mp.	160
5	FGID.mp.	466
6	gastro * disorder.mp.	1475
7	functional gut.mp.	166
8	3 or 4 or 5 or 6 or 7	4316
9	2 and 8	160
10	child *.mp. or Child/	2,503,644
11	9 and 10	55
12	p *ediatric.mp.	371,166
13	10 or 12	2,579,625
14	9 and 13	57
15	Adolescent/ or adolescen *.mp.	2,153,986
16	10 or 12 or 15	3,677,129
17	9 and 16	62

*: search truncation symbol.

**Table A2:** EMBASE.

Search Line	Search Statement	Results
1	Rome IV.mp.	1062
2	limit 1 to yr = “2016 -Current”	1042
3	functional gastro *.mp.	5412
4	GI disorder.mp.	397
5	FGID.mp.	1031
6	gastro * disorder.mp.	2902
7	functional gut.mp.	270
8	3 or 4 or 5 or 6 or 7	7847
9	child *.mp. or child/	2,774,584
10	p *ediatric.mp.	573,340
11	adolescent/ or adolescence/ or adolescen *.mp.	1,636,092
12	9 or 10 or 11	3,615,317
13	2 and 8 and 12	130

*: search truncation symbol.

**Table A3:** CINAHL.

Search Line	Search Statement	Results
1	Rome IV	
2	Child * OR p *ediatric OR adolescen *	
3	FGID OR functional gastro * OR gastro * disorder OR functional gut	
4	1 and 2 and 3	37

*: search truncation symbol.

**Table A4:** PSYCHINFO.

Search Line	Search Statement	Results
1	Rome IV.mp.	11
2	limit 1 to yr = “2016 -Current”	11
3	functional gastro *.mp.	376
4	GI disorder.mp.	31
5	FGID.mp.	58
6	gastro * disorder.mp.	164
7	functional gut.mp.	6
8	3 or 4 or 5 or 6 or 7	471
9	child *.mp.	821,999
10	exp Pediatrics/ or p *ediatric.mp.	49,253
11	Adolescen *.mp.	478,850
12	9 or 10 or 11	1,094,309
13	2 and 8 and 12	2

*: search truncation symbol.

**Table A5:** Joanna Briggs.

Search Line	Search Statement	Results
1	Rome IV AND (child * OR adolescen * OR p *ediatric)	601
2	limit 1 to yr = “2016 -Current”	289
3	Rome IV.mp.	4
4	limit 3 to yr = “2016 -Current”	4
5	functional gastro *.mp.	10
6	GI disorder.mp.	0
7	FGID.mp.	0
8	gastro * disorder.mp.	3
9	functional gut.mp.	0
10	5 or 6 or 7 or 8 or 9	13
11	4 and 10	1
12	child *.mp. or Child/	2024
13	11 and 12	1
14	p *ediatric.mp.	874
15	12 or 14	2210
16	11 and 15	1
17	Adolescent/ or adolescen *.mp.	683
18	12 or 14 or 17	2281
19	11 and 18	1

*: search truncation symbol.

**Table A6:** PROQUEST.

Search Line	Search Statement	Results
1	(child * OR p *ediatric OR adolescent *)	
2	(Rome NEAR/1 IV)	
3	(FGID OR functional NEAR/1 gastro * OR functional NEAR/1 gut OR gastro * NEAR/1 disorder OR GI NEAR/1 disorder)	
4	1 and 2 and 3	100

*: search truncation symbol.

**Table A7:** SCOPUS.

Search Line	Search Statement	Results
1	(TITLE-ABS-KEY ( rome w/1 iv ) )	
2	(child * OR adolescen * or p *ediatric)	
3	(fgid OR gi w/1 disorder OR gastro * w/1 disorder OR functional w/1 gut OR functional w/1 gastro *)	
4	1 and 2 and 3	31

*: search truncation symbol.

**Table A8:** Cochrane (Cochrane library publication date from January 2016 to March 2021).

Search Line	Search Statement	Results
1	child *	182,167
2	p *ediatric	53,365
3	Adolescen *	142,978
4	MeSH descriptor (child) explode all trees	56,688
5	MeSH descriptor (adolescent) explode all trees	104,818
6	Rome IV	494
7	FGID	61
8	“Functional gastro *”	11
9	“Gastro * disorder”	0
10	GI disorder	55
11	“Functional gut”	36
12	(#1 OR #2 OR #3 OR #4 OR #5)	281,555
13	(#7 OR #8 OR #9 OR #10 OR #11)	160
14	#12 AND #6 AND #13	3

*: search truncation symbol.

**Table A9:** OPEN GREY.

Search Line	Search Statement	Results
1	Functional + gastrointestinal + disorders	10
2	FGID	0
3	Rome + IV + gastrointestinal	0

## References

[1] Lewis M.L., Palsson O.S., Whitehead W.E., van Tilburg M.A. (2016). Prevalence of Functional Gastrointestinal Disorders in Children and Adolescents. J. Pediatr..

[2] Sperber A.D., Bangdiwala S.I., Drossman D.A., Ghoshal U.C., Simren M., Tack J., Whitehead W.E., Dumitrascu D.L., Fang X., Fukudo S. (2021). Worldwide Prevalence and Burden of Functional Gastrointestinal Disorders, Results of Rome Foundation Global Study. Gastroenterology.

[3] Caplan A., Walker L., Rasquin A. (2005). Development and Preliminary Validation of the Questionnaire on Pediatric Gastrointestinal Symptoms to Assess Functional Gastrointestinal Disorders in Children and Adolescents. J. Pediatr. Gastroenterol. Nutr..

[4] Jarrett M., Heitkemper M., Czyzewski D.I., Shulman R. (2003). Recurrent Abdominal Pain in Children: Forerunner to Adult Irritable Bowel Syndrome?. J. Spec. Pediatr. Nurs..

[5] Hyams J.S., Di Lorenzo C., Saps M., Shulman R.J., Staiano A., van Tilburg M. (2016). Childhood Functional Gastrointestinal Disorders: Child/Adolescent. Gastroenterology.

[6] Benninga M.A., Nurko S., Faure C., Hyman P.E., Roberts I.S.J., Schechter N.L. (2016). Childhood Functional Gastrointestinal Disorders: Neonate/Toddler. Gastroenterology.

[7] Page M.J., McKenzie J.E., Bossuyt P.M., Boutron I., Hoffmann T.C., Mulrow C.D., Shamseer L., Tetzlaff J.M., Akl E.A., Brennan S.E. (2021). The PRISMA 2020 statement: An updated guideline for reporting systematic reviews. BMJ.

[8] Loney P.L., Chambers L.W., Bennett K.J., Roberts J.G., Stratford P. (1998). Critical appraisal of the health research literature: Prevalence or incidence of a health problem. Chronic Dis. Can..

[9] Aydemir Y., Carman K.B., Yarar C. (2020). Screening for functional gastrointestinal disorders in children with epilepsy. Epilepsy Behav..

[10] Beser O., Cokugras F., Vandenplas Y. (2019). Prevalence and clinical characteristics of functional gastrointestinal disorders in infants [abstract]. J. Pediatr. Gastroenterol. Nutr..

[11] Chanis R., Velasco-Benitez C. (2019). Prevalence of functional gastrointestinal disorders in panamanian infants under 12 months: Comparison between the Rome III and Rome IV Criteria [abstract]. J. Pediatr. Gastroenterol. Nutr..

[12] Chew K.S., Em J.M., Koay Z.L., Jalaludin M.Y., Ng R.T., Lum L.C.S., Lee W.S. (2020). Low prevalence of infantile functional gastrointestinal disorders (FGIDs) in a multi-ethnic Asian population. Pediatr. Neonatol..

[13] Chikunov V., Ilenkova N. (2019). Prevalence of functional gastrointestinal disorders in Russian children [abstract]. Turk. J. Gastroenterol..

[14] Ekinci R.M.K., Balcı S., Akay E., Tumgor G., Dogruel D., Altintas D.U., Yilmaz M. (2019). Frequency of functional gastrointestinal disorders in children with familial Mediterranean fever. Clin. Rheumatol..

[15] Ekinci R.M.K., Balcı S., Mart O.O., Tumgor G., Yavuz S., Celik H., Dogruel D., Altintas D.U., Yilmaz M. (2018). Is Henoch–Schönlein purpura a susceptibility factor for functional gastrointestinal disorders in children?. Rheumatol. Int..

[16] Játiva-Mariño E., Rivera-Valenzuela M.G., Velasco-Benitez C.A., Saps M. (2019). The prevalence of functional constipation in children was unchanged after the Rome IV criteria halved the diagnosis period in Rome III. Acta Paediatr..

[17] Llanos-Chea A., Velasco-Benitez C., Saps M. (2019). Inter-observer reliability for stool consistency between the bristol stool scale and the Brussels Infant and Toddler Stool Scale (BITSS) when using Rome IV criteria in younger children [abstract]. J. Pediatr. Gastroenterol. Nutr..

[18] Ozdemir M., Beser O. (2018). Study on determination of functional gastrointestinal diseases in infants who were received the general pediatric outpatient clinic by using Rome Iv diagnostic criteria [abstract]. J. Pediatr. Gastroenterol. Nutr..

[19] Robin S.G., Keller C., Zwiener R., Hyman P.E., Nurko S., Saps M., Di Lorenzo C., Shulman R.J., Hyams J.S., Palsson O. (2018). Prevalence of Pediatric Functional Gastrointestinal Disorders Utilizing the Rome IV Criteria. J. Pediatr..

[20] Saps M., Velasco-Benitez C.A., Langshaw A.H., Ramírez-Hernández C.R. (2018). Prevalence of Functional Gastrointestinal Disorders in Children and Adolescents: Comparison between Rome III and Rome IV Criteria. J. Pediatr..

[21] Saps M., Velasco-Benitez C.A., Valdes L.F., Mejia J., Villamarin E., Moreno J., Ramirez C., González M.J., Vallenilla I., Falcon A.C. (2020). The impact of incorporating toilet-training status in the pediatric Rome IV criteria for functional constipation in infant and toddlers. Neurogastroenterol. Motil..

[22] Steutel N.F., Zeevenhooven J., Scarpato E., Vandenplas Y., Tabbers M.M., Staiano A., Benninga M.A. (2020). Prevalence of Functional Gastrointestinal Disorders in European Infants and Toddlers. J. Pediatr..

[23] Velasco-Benitez C., Campeon-Cruz V., Duenas-Armendariz A. (2018). Reproducibility of the Rome IV questionnaire for pediatric digestive symptoms in Spanish for functional gastrointestinal disorders in schoolchildren and adolescents from Colombia, South America [abstract]. J. Pediatr. Gastroenterol. Nutr..

[24] Velasco-Benitez C., Villamarin-Betancourt E., Mejia-Lopez J. (2019). Risk factors in children under 4 years of age with functional gas-trointestinal disorders according to the Rome IV criteria [abstract]. J. Pediatr. Gastroenterol. Nutr..

[25] Velasco-Benitez C.A., Axelrod C., Gutierrez S., Saps M. (2020). The Relationship Between Prematurity, Method of Delivery, and Functional Gastrointestinal Disorders in Children. J. Pediatr. Gastroenterol. Nutr..

[26] Velasco-Benítez C.A., Gómez-Oliveros L.F., Rubio-Molina L.M., Tovar-Cuevas J.R., Saps M. (2020). Diagnostic Accuracy of the Rome IV Criteria for the Diagnosis of Functional Gastrointestinal Disorders in Children. J. Pediatr. Gastroenterol. Nutr..

[27] Vlad R., Dijmarescu I., Smadeanu R. (2019). Functional gastrointestinal disorders, up to date topic. Applying Rome iv criteria to a Romanian cohort of young children [abstract]. J. Pediatr. Gastroenterol. Nutr..

[28] Zeevenhooven J., Biesbroek A., Schappin R. (2019). Functional gastrointestinal disorders and behavioral problems in adolescents with a history of infant colic [abstract]. Gastroenterology.

[29] Koppen I.J.N., Nurko S., Saps M., Di Lorenzo C., Benninga M.A. (2017). The pediatric Rome IV criteria: What’s new?. Expert Rev. Gastroenterol. Hepatol..

[30] Ferreira-Maia A.P., Matijasevich A., Wang Y.-P. (2016). Epidemiology of functional gastrointestinal disorders in infants and toddlers: A systematic review. World J. Gastroenterol..

[31] Boronat A., Ferreira-Maia A.P., Matijasevich A., Wang Y.-P. (2017). Epidemiology of functional gastrointestinal disorders in children and adolescents: A systematic review. World J. Gastroenterol..

[32] Diederen K., Hoekman D.R., Hummel T.Z., De Meij T.G., Koot B.G.P., Tabbers M.M., Vlieger A.M., Kindermann A., Benninga M.A. (2016). The prevalence of irritable bowel syndrome-type symptoms in paediatric inflammatory bowel disease, and the relationship with biochemical markers of disease activity. Aliment. Pharmacol. Ther..

[33] Halpin S.J., Ford A. (2012). Prevalence of Symptoms Meeting Criteria for Irritable Bowel Syndrome in Inflammatory Bowel Disease: Systematic Review and Meta-Analysis. Am. J. Gastroenterol..

[34] Silvester J.A., Graff L.A., Rigaux L., Bernstein C.N., Leffler D.A., Kelly C.P., Walker J.R., Duerksen D.R. (2017). Symptoms of Functional Intestinal Disorders Are Common in Patients with Celiac Disease Following Transition to a Gluten-Free Diet. Dig. Dis. Sci..

[35] Kovacic K., Chelimsky T.C., Sood M.R., Simpson P., Nugent M., Chelimsky G. (2014). Joint Hypermobility: A Common Association with Complex Functional Gastrointestinal Disorders. J. Pediatr..

[36] Phatak U.P., Pashankar D.S. (2014). Prevalence of functional gastrointestinal disorders in obese and overweight children. Int. J. Obes..

[37] Saps M., Sansotta N., Bingham S., Magazzu G., Grosso C., Romano S., Pusatcioglu C., Guandalini S. (2017). Abdominal Pain-Associated Functional Gastrointestinal Disorder Prevalence in Children and Adolescents with Celiac Disease on Gluten-Free Diet: A Multinational Study. J. Pediatr..

[38] Velasco-Benítez C.A., Ruiz-Extremera Á., Saps M. (2019). Case–control study on generalised joint hypermobility in schoolchildren with functional gastrointestinal disorders according to Rome IV criteria in Spanish. An. Pediatr..

[39] Pinheiro L.C., McFatrich M., Lucas N., Walker J.S., Withycombe J.S., Hinds P.S., Sung L., Tomlinson D., Freyer D.R., Mack J.W. (2017). Child and adolescent self-report symptom measurement in pediatric oncology research: A systematic literature review. Qual. Life Res..

[40] Waters E., Stewart-Brown S., Fitzpatrick R. (2003). Agreement between adolescent self-report and parent reports of health and well-being: Results of an epidemiological study. Child Care Health Dev..

