# Supplementary material for: Systematic Review of Pediatric Functional Gastrointestinal Disorders (Rome IV Criteria)
Source: J Clin Med. 2021 Oct 29;10(21):5087. doi: 10.3390/jcm10215087 (PMC8585107; doi:10.3390/jcm10215087)
Supplement: Supplementary file 1 [file jcm-10-05087-s001.zip › AVR_Table S1.pdf]

Table S1: Risk of bias assessment for included articles

✓ = One point scored, ● = Half point scored, × = Zero points scored

[a] = full article, [b] = conference abstract

	Validity of study methods					Results interpretation		Applicability of results	
**First Author**	**Population sampling**	**Location sampling**	**Sample size**	**Standard Measures**	**Unbiased assessor**	**Confidence intervals**	**Complete reporting**	**Study subjects**	**Quality Score**
Aydemir [12]	×	×	×	✓	✓	✓	✓	×	4[a]
Beser [13]	✓	×	✓	✓	✓	×	×	×	4[b]
Chanis [15]	✓	×	×	✓	✓	×	✓	✓	5[b]
Chew [16]	✓	●	✓	✓	✓	✓	✓	✓	7.5[a]
Chikunov [17]	✓	×	✓	✓	×	×	✓	×	4[b]
Ekinci [18]	×	×	×	✓	✓	×	✓	✓	4[a]
Ekinci [19]	×	×	✓	✓	×	×	✓	✓	4[a]
Jativa-Marino [20]	✓	✓	✓	●	✓	×	×	✓	5.5[a]
Llanos-Chea [21]	✓	●	✓	✓	✓	×	×	×	4.5[b]
Ozdemir [22]	✓	×	✓	✓	×	×	×	×	3[b]
Robin [23]	●	✓	✓	✓	✓	×	✓	✓	6.5[a]
Saps [24]	✓	✓	✓	✓	✓	×	✓	✓	7[a]
Saps [25]	✓	×	✓	✓	✓	×	×	✓	5[a]
Steutel [26]	✓	●	✓	✓	✓	✓	✓	✓	7.5[a]
Velasco-Benitez [27]	✓	×	✓	✓	✓	×	×	✓	5[b]
Velasco-Benitez [28]	✓	×	✓	✓	✓	×	×	✓	5[b]
Velasco-Benitez [29]	✓	✓	✓	✓	✓	×	✓	✓	7[a]
Velasco-Benítez [30]	✓	✓	✓	✓	✓	×	✓	✓	7[a]
Vlad [31]	✓	●	✓	●	×	×	✓	×	4[b]
Zeevenhooven [32]	×	×	×	✓	✓	✓	✓	✓	5[b]

Population sampling - Is the population sampling method appropriate for the research question?
Scores: Random or whole population sampling = 1 point, selecting respondents within these methods = 0.5 point
Location sampling - Is the sampling location appropriate for the general population?
Scores: Regional sampling = 1 point, Sampling from more than one general health clinic = 0.5 point
Sample size adequate - Is the sample size adequate? Determined by previous work among children showing FGID prevalence at approximately 30% [8, 9], therefore, a minimal sample size of 150 could get adequate precision of FGID prevalence.
Scores: Sample size greater than 150 = 1 point
Standard measures - Are objective criteria used for measuring the health outcome, with appropriate validation?
Scores: English Rome IV criteria, or translation carried out with appropriate methodology = 1 point, translated Rome IV if methodology not stated or validation not carried out = 0.5 point.
Un-biased assessor - Is the health outcome measured in a non-biased fashion?
*Scores***:** Study reported who completed Rome IV = 1 point
Confidence intervals - Are the results presented with confidence intervals for their estimates?
Scores: Confidence intervals reported = 1 point
Complete reporting - Is there complete reporting of all results?
Scores: Complete reporting of data (including ‘zero’ if no participants) = 1 point
Study subjects - Are study subjects sufficiently described?
Scores: Reporting age and gender distribution for study population = 1 point
