# Supplementary figures and images for: Systematic Review of Pediatric Functional Gastrointestinal Disorders (Rome IV Criteria)
Source: J Clin Med. 2021 Oct 29;10(21):5087. doi: 10.3390/jcm10215087 (PMC8585107; doi:10.3390/jcm10215087)

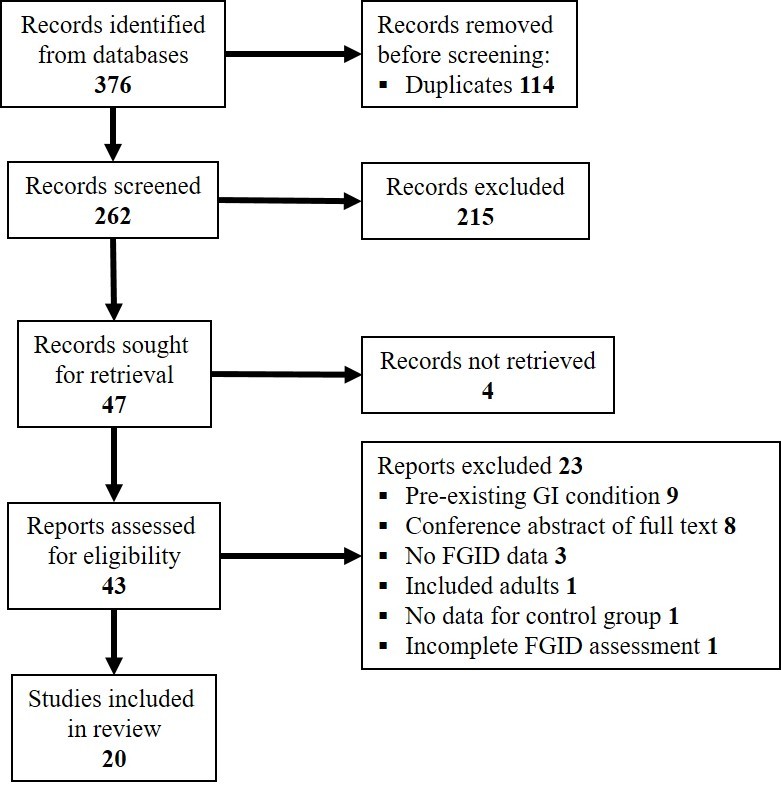

Supplement: Supplementary file 1 [file jcm-10-05087-s001.zip › AVR_Figure S1.jpg]

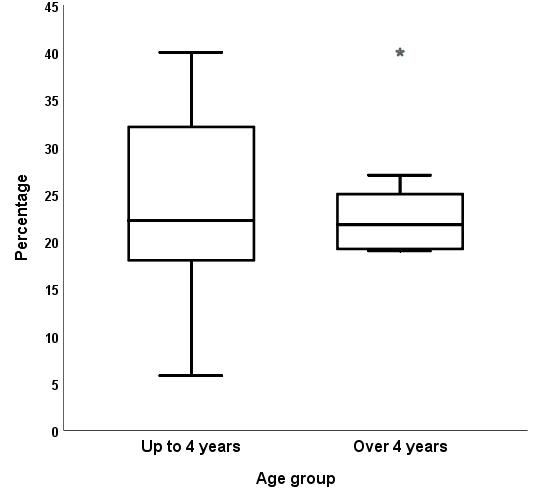

Supplement: Supplementary file 1 [file jcm-10-05087-s001.zip › AVR_Figure S2.jpg]
